# On the Mechanical Treatment of Diseases of the Hip-Joint

**Published:** 1873-12

**Authors:** 


*On the Mechanical Treatment of Diseases of the Hip-Joint.* By Charles Fayette Taylor, M. D. New York: Wm. Wood & Co., 1873; Buffalo: H. H. Otis.

This is a little book giving the authors views and the result of his observations in the mechanical treatment of Hip-Joint Disease

With a large experience in the treatment of this class of diseases, his observations have been such as will be confirmed by those in the habit of treating these affections.

The indications for mechanical treatment are:

"First—To relieve the pressure in the joint due to muscular contraction, by temporarily destroying the muscular irritability and contractibility.

Second—To protect the joint from weight and concussion."

"Motion in the joint without pressure is not injurious but is highly beneficial."

The essay gives a full description of Dr. Taylor's instrument and its mode of application. The above indications seem to be fully met by its application.

There are no appliances, however, which are suited for the treatment of Hip-joint Disease, which are not applied with care by experienced hands, and which are not patiently and carefully watched, both by the physician and those having the patient in charge. Patient perseverance should be the motto in treating Hip-joint Disease.

